# Poster Session II - A235 PSYCHOLOGICAL STRESS IMPAIRS MUCOSAL GUT BARRIER INTEGRITY AND LEADS TO LOSS OF HOST CONTROL OF CROHN’S DISEASE-ASSOCIATED ADHERENT-INVASIVE *E. COLI*

**DOI:** 10.1093/jcag/gwaf042.235

**Published:** 2026-02-13

**Authors:** M Zangara, H Sham, K Choi, B Vallance, B Coombes

**Affiliations:** McMaster University, Hamilton, ON, Canada; The University of British Columbia, Vancouver, BC, Canada; The University of British Columbia, Vancouver, BC, Canada; The University of British Columbia, Vancouver, BC, Canada; McMaster University, Hamilton, ON, Canada

## Abstract

**Background:**

Crohn’s disease (CD) arises from complex interactions between host genes, intestinal microbiome, and environmental triggers. Within the microbiota compartment, adherent-invasive *E. coli* (AIEC) is strongly associated with ileal CD. Using a preclinical model, we previously showed that restraint-induced psychological stress suppresses host IL-22 production via apoptosis of CD45^+^CD90^+^ cells, enabling AIEC expansion throughout the intestine.

**Aims:**

To determine how psychological stress compromises mucosal barrier integrity in the context of AIEC infection.

**Methods:**

Wild-type C57Bl/6 mice were infected with AIEC strain LF82 and subjected to psychological stress via overnight restraint. Ileal tissue was collected post-stress for molecular and histological analyses, and primary organoid cultures were generated to assess the regenerative potential of the stem cell compartment.

**Results:**

Psychological stress significantly reduced Paneth cell (PC) function, as evidenced by reduced IL-22-induced antimicrobial peptides (AMPs) expression. PCs isolated from stressed mice showed reduced antibacterial activity (*p*<0.01) and histological analysis revealed increased immune cell infiltration (*p*<0.01), consistent with impaired microbial control. Although gross epithelial integrity remained intact, the number of proliferating Ki67^+^ epithelial cells was markedly reduced in stressed mice (*p*<0.0001). This was accompanied by decreased expression of stem cell markers *Lgr5* (*p*<0.05) and *Fgfbp1* (*p*<0.001) and *Wnt3* (*p*<0.05). Ileal crypts isolated from stressed mice generated significantly fewer organoids (*p*<0.0001) compared to unstressed controls. Expression of terminal differentiation markers for PCs (*Sox9*, *p*<0.05) and Goblet cells (*Klf4*, *p*<0.001), were also reduced post-stress.

**Conclusions:**

Psychological stress diminishes the regenerative capacity of ileal stem cells, disrupting epithelial cell lineage differentiation and mucosal defense mechanisms. These stress-induced impairments weaken antimicrobial defenses and disrupt mucosal homeostasis, permitting enhanced colonization by CD-associated AIEC. The maladaptive response in this study helps to define the exacerbating role of stress in CD pathogenesis and highlights an avenue in which to focus therapeutic research efforts that targets mucosal barrier integrity.

**Funding Agencies:**

CCC, CIHR

